# Supplementary material for: Algorithm for diagnosis of early Schistosoma haematobium using prodromal signs and symptoms in pre-school age children in an endemic district in Zimbabwe
Source: PLoS Negl Trop Dis. 2021 Aug 2;15(8):e0009599. doi: 10.1371/journal.pntd.0009599 (PMC8360514; doi:10.1371/journal.pntd.0009599)
Supplement: Attachment — Submitted filename: Reviewer_report_PLOS_NTD.docx [file pntd.0009599.s001.docx]

**Algorithm for Diagnosis of Early *Schistosoma haematobium*** 1 **using Prodromal Signs and**

**Symptoms in Pre-school Age Children in an Endemic District in Zimbabwe**

Dear Editor

Thank you for giving me this opportunity to read an interesting work on the topic of schistosomiasis in pre-school children, an important but neglected in term of control activities. Interestingly, the study objective was to find a simple tool to be used at community level and primary health system to identify pre-school children at risk or infected with *S. haematobium*. The study is valid owing the lack of pediatric PZQ and is worth for publication and I have the following observations: -

**Background**

- The use of the word infestation versus infection? Infestation is for ectoparasites and infection is for endoparasites
- Background is too long and some information presented here are good for the discussion. You can find that, similar information is presented in the discussion section

Ethics and ethical statement

- A detail description of how children with these other diseases diagnosed in the study were managed is required
- Issues of confidentiality and treatment of PASC needs to be described
- What happens to children who tested positive on the first and second follow-up?

Data Analysis

- I find some 95% confidence interval have very wide margins, I proposed the use of GLM to generate OR and aOR.

Results

- Present a table of logistic regression with bivariate and multivariate results for readers to review it
- Put a risk scores in a table form and describe this in a data analysis section

Discussion

- Some information are repetition of what has been presented in the background section
