# Supplementary material for: Algorithm for diagnosis of early Schistosoma haematobium using prodromal signs and symptoms in pre-school age children in an endemic district in Zimbabwe
Source: PLoS Negl Trop Dis. 2021 Aug 2;15(8):e0009599. doi: 10.1371/journal.pntd.0009599 (PMC8360514; doi:10.1371/journal.pntd.0009599)
Supplement: Attachment — Submitted filename: Rebuttal Table responses.docx [file pntd.0009599.s003.docx]

Point-by-point responses to the comments raised by PLOS NTD reviewers

| **Reviewer 2 comments** | | |
| --- | --- | --- |
| **Reviewer’s Comments** | **Response** | **Section** |
| The use of the word infestation versus infection? Infestation is for ectoparasites and infection is for endoparasites | Thank you for your comment, it is noted and corrected in line 78 and 81 | **Background** |
| Background is too long and some information presented here are good for the discussion. You can find that, similar information is presented in the discussion section | Thank you for the comment we corrected it by removing repeated information in the following lines: 73-79 and 118-122 |  |
| Ethics and ethical statement   1. A detail description of how children with these other diseases diagnosed in the study were managed is required. 2. Issues of confidentiality and treatment of PASC needs to be described 3. What happens to children who tested positive on the first and second follow-up? | -Thank you for the suggestion, and we have added information on the treatment in line 176 -179  -Thank you for your comment, a statement on confidentiality has been added in line:176 and 182-183.  -Children who tested positive were treated accordingly in line 183 and 157. | **Methodology** |
| Data Analysis   1. I find some 95% confidence interval have very wide margins, I proposed the use of GLM to generate OR and aOR. | -Thank you for the comment we have added a Table with GLM P-values in Table 5. |  |
| Results   1. Present a table of logistic regression with bivariate and multivariate results for readers to review it 2. Put a risk scores in a table form and describe this in a data analysis section | -Thank you for the suggestion, we added a column with bivariate values to Table 3.  -Thank you for the suggestion, we did as advised line 317 – 319 and in Table 5. | Results |
| 1.Some information are repetition of what has been presented in the background section | Thank you for the suggestion, we removed repeated information from the introduction | Discussion |
| **Reviewer 1** | | |
| **Reviewer’s Comments** | **Response** | **Section** |
| 1. Abstract, lines 25-26: please state the timing of the follow-up visits and not just "for a period of 6 months". 2. Abstract, lines 43-44: please state the comparator (i.e., vs. non-ESI?). | -Thank you for the comment we have corrected in line 26  ‘the study ran from July 2019-December 2019’  -Thank you for your comment, it is corrected lines 45-48 | Abstract |
| 1. Introduction, line 79: "may manifest" not "manifests". 2. Introduction, line 86: I would specify that "parasitological diagnosis" refers to egg detection via microscopy. 3. Introduction, lines 110-111: this statement is somewhat misleading. Praziquantel is not very effective in treating "initial stages of infection" prior to development of adult worms. This statement should be qualified to acknowledge this. | -Thank you for the correction, we corrected in line 87  -Thank you for the addition: parasitological diagnosis was defined in line 93  -Thank you for your comment, and we agree with reviewer’s point. in this article we focus on early signs and symptoms of schistosomiasis as an entity on its own. We did not look at praziquantel efficacy, but this would be an appropriate next step. | Introduction |
| 1. Methods, line 125: "temperate" not "temperament | -Thank you for the correction, it is corrected in line 130 | Methods |
| 2. Methods, line 134: the authors state that they included children at "high risk" but do not define how this was determined. Please clarify. | High risk was determined by virtue of the study site having the highest prevalence of schistosomiasis in the country Clarity was added in line 147 - 158 |  |
| 3. Lines 271-273: it would be useful to have a table showing the proportions of both ESI and non-ESI children with the different signs and symptoms, rather than just stating the proportions for the ESI participants. It would help visualize the comparison | - Thank you for your comment, the results in Table 2 are comparing *S. haematobium* infected participants at the different visits with the participants classified as ESI. |  |
| 4. Methods, line 146: please describe the TOrCHeS screen that was conducted. Were serological assays performed? If so, which ones? | - Thank you for your comment, method used to test ToRCHeS is described in the Methods section under subheading Blood processing and analysis lines 221-235 |  |
| 5. Methods, line 147: why was a Widal test done as part of screening for this study and what is the relevance as an inclusion criterion? | - Thank you for your comment, typhoid is endemic in the area of the study. Typhoid manifests differently in the pediatric population thus we did not want to attribute signs and symptoms due to typhoid as those for schistosomiasis. Of course Widal test is not very accurate but it is the best we could do to cover typhoid as a limitation. |  |
| 6. Methods, line 181: I would just use the title, "microhaematuria screen" rather than "urinary morbidity" since morbidity isn't really being evaluated | Thank you for your comment, correction was made in line 193 |  |
| 7. Methods, line 202: should be "blood urea nitrogen" concentration, not just "urea". | Thank you for your comment, we assayed the whole urea compound not just BUN |  |
| 8. Methods, line 205: please include the units for haemoglobin values. | Thank you for your comment, it is corrected lines 215-218 |  |
| 9. Methods, line 209: which hepatitides were evaluated and which antigens/antibodies? | Thank you for your comment, we clarified in line 222. We measured hepatitis A, B and C IgG and IgM. |  |
| 10. Methods, line 210: "Giemsa" not "geimsa". | Thank you for the comment, we corrected in line 223 |  |
| 1. Table 1: under the "S. haematobium negative" sub header there are two columns but it is unclear what the difference is between the two. | Thank you for your comment, the first column is 2^nd^ visit and the second column is the third visit | Results |
| 2. Table 2: please add the time-points (e.g., 4 weeks, 16 weeks) to the columns. Also, it would be helpful to include mean egg counts rather than just the intensity category. | Thank you for your comment, we have corrected in line 282 and in Table 2 under infection intensity. |  |
| 3. Table 3 (the second one): what does the "**" superscript refer to? It isn't defined. 4. Table 3: as a footnote, please indicate what the AOR is in comparison to (i.e., children with no signs or symptoms). | Thank you for your comment, we have removed the mark, however it meant to show a very strong association  The adjusted odds ratio are comparing prodromal signs and symptoms amongst *S. haematobium* infected participants to those who are negative |  |
| 1. The authors mention that a questionnaire was administered to parents/guardians of the children, but the content of this questionnaire isn't elucidated. It would be best if a brief description of the type of questions could be included, or even better, include the questionnaire as supplemental material. | Thank you for your comment, additional material has been added although the outcome from the questionnaire and clinical observation is summarized in Table 3 and 5. |  |
| 2. The main criticism of this paper is the conclusion that the algorithm could be used to identify children who could be treated early to prevent chronic schistosomiasis. However, the authors only passingly make reference to the stage-specific effectiveness of praziquantel, when in fact this could be a major barrier to using this algorithm to guide therapy in endemic areas. Praziquantel is effective against cercariae and very young schistosomula but not against older schistosomula, which is exactly the stage of development that the worms infecting the children identified by the algorithm are likely at. More discussion of this limitation is warranted. | Thank you for your comment. It is duly noted and we agree with the reviewer that praziquantel is not effective in the early stages. We are hopeful that with changing times we will be able to identify an early therapeutic drug for schistosomiasis. However, our algorithm is meant to be a screening tool before doing urine filtration in resource limited settings. As in our case, we use urine dipstick as a screening tool before sending the patient to a hospital with lab technicians (which are very few to cover the entire country). Thus identifying the early schistosomiasis signs and symptoms is of paramount importance irrelevant of the praziquantel efficacy. | Conclusion |
| **Reviewer 3** | | |
| **Reviewer’s Comments** | **Response** | **Section** |
| **Abstract**  It is well written and clear.  **Key words**  PSAC should be written first in full before the abbreviation | Thank you for the comment, much appreciated  Comment noted and corrected in line 56 and 117 -118. | Abstract |
| **Introduction**  It is quite detailed but also has errors   1. Line number 78, the citation (10-(10) is confusing | Thank you for the comment we have corrected the error, now the section has been deleted because there was repetition of the concept in the discussion section | Introduction |
| **Methodology**  The design and methods are clear   1. Line 132, the authors say July- December when there is highest schistosomiasis transmission, there is no evidence to that effect. Any reference? | Thank you for the comment, we have added a reference in which the seasons were studied in relation to schistosomiasis in Zimbabwe (Rhodesia) reference [17]; in line 136. | Methodology |
| **Results**  They are well presented   1. Table 1, how was the contaminated water identified? 2. Figures 2 and 3 should have the word ‘ Figure’ written bellow the actual figure and not on top | 1. Contaminated water was identified from previous studies in the area which noted high transmission water sources with fresh water snail in abundance, we have added the reference.  2. Thank you for the comment, we have corrected the mistake. | Results |
| **Discussion**  It is well written and quite detailed   1. Line 382, please add ‘in’ after the word ‘difficulties’ | Thank you for the comment, we have corrected the error line 411 | Discussion |
| **Conclusion**  The study is well concluded. Please attend to the question bellow.   1. Inguinal lymphadenopathy is common in people with injuries / infection to the lower extremities, especially in children who even like playing with bare feet. How reliable would inguinal lymphadenopathy be a sign of schistosomiasis? | Thank you for the comment. We agree with the reviewer inguinal lymphadenopathy is common in children with injuries of the lower limbs. However the inguinal lymphadenopathy being mentioned in this context were after a full body examination and no injuries noted as described in our previous paper titled: Clinical Morbidity associated with children under the age of five from a schistosomiasis endemic area in Zimbabwe  In the paper odds of having inguinal lymphadenopathy in a schistosomiasis positive PSAC were 99 times and attributable fraction was found to be 68.4%. | Conclusion |
|  |  |  |
