# Supplementary material for: Algorithm for diagnosis of early Schistosoma haematobium using prodromal signs and symptoms in pre-school age children in an endemic district in Zimbabwe
Source: PLoS Negl Trop Dis. 2021 Aug 2;15(8):e0009599. doi: 10.1371/journal.pntd.0009599 (PMC8360514; doi:10.1371/journal.pntd.0009599)
Supplement: Attachment — Submitted filename: Responses to Reviewer comments final.docx [file pntd.0009599.s002.docx]

**Responses to reviewers**

**Background**

1. The use of the word infestation versus infection? Infestation is for ectoparasites and infection is for endoparasites

**Responses**

Thank you for your comment, it is noted and corrected in line 76 and79

‘The fever occurs from 4 weeks post infection and the symptoms include chills… The chronic phase of *S. haematobium* infection begin 12-16 weeks post infection’

2. Background is too long and some information presented here are good for the discussion. You can find that, similar information is presented in the discussion section

**Responses**

thank you for the comment we corrected it by removing repeated information in the following lines: 73-79 and 99-101

Ethics and ethical statement

1. A detail description of how children with these other diseases diagnosed in the study were managed is required.

**Responses**

Thank you for the suggestion, and we have added information on the treatment in line 153

‘Children diagnosed of other diseases were managed appropriately and according to World Health Organisation (WHO) standards. For confidentiality purposes all participants were allocated a number and underwent individual counselling and private treatment depending on their schistosomiasis infection status.’

2. Issues of confidentiality and treatment of PASC needs to be described

**Responses**

Thank you for your comment, a statement on confidentiality has been added on line:183

3. What happens to children who tested positive on the first and second follow-up?

**Responses**

Children who tested positive were treated accordingly in line 153 – 157.

Data Analysis

1. I find some 95% confidence interval have very wide margins, I proposed the use of GLM to generate OR and aOR.

**Responses**

Thank you for the comment we have provided additional information line 214 – 217 and in Table 3 with GLM and P-values

Results

1.Present a table of logistic regression with bivariate and multivariate results for readers to review it

**Responses**

Thank you for the suggestion, we added a column with bivariate values in Table 3.

2. Put a risk scores in a table form and describe this in a data analysis section

**Responses**

Thank you for the suggestion, we did as advised; presented Table 5 with Risk Scores, additionally defined CHARI and explained in line 275 – 280

‘A screening tool named CHARI was created, where C = Contaminated water exposure; H = Hot body or haematuria; A = Abdominal distension; R = Rash after water exposure/Recurring fungal infections; and I = Inguinal lymph nodes enlarged. Using Youden’s index determination for bootstrapped population and the alternative approach of splitting the receiver operating characteristic (ROC) into two curves of sensitivity and specificity, we retrieved an inconclusive zone spreading from 4-8 and 4.1-8.4, respectively. we rounded the grey zone score to 4-8. The rate of confirmed schistosomiasis increased significantly from 0% (0/69) for score ≤3 to 59% (39/66) for score ≥9, 33% (68/204) participants were in the grey zone of 4-8 and confirmed cases were 0.04% (3/68).’

Discussion

1.Some information are repetition of what has been presented in the background section

**Responses**

Thank you for the suggestion, we removed repeated information from the introduction and the sections are shown in tracked changes.

Responses.

Graphs have been provided in .tiff format and also the table shave been included in the revised manuscript instead of standing alone as a file.
